# Predicting fracture risk after non-recent high risk fracture: improving accuracy with simple modifiers to FRAX

**DOI:** 10.1093/jbmr/zjag049

**Published:** 2026-03-11

**Authors:** Carrie Ye, Suzanne N Morin, Lisa M Lix, Eugene V McCloskey, Helena Johansson, Nicholas C Harvey, John A Kanis, William D Leslie

**Affiliations:** University of Alberta, Edmonton, Canada; McGill University, Montreal, Canada; University of Manitoba, Winnipeg, Canada; Centre for Metabolic Bone Diseases, University of Sheffield Medical School, Sheffield, UK; MRC Versus Arthritis Centre for Integrated research in Musculoskeletal Ageing, Department of Oncology & Metabolism, University of Sheffield, Sheffield UK; Centre for Metabolic Bone Diseases, University of Sheffield Medical School, Sheffield, UK; Sahlgrenska Osteoporosis Centre, Institute of Medicine, Sahlgrenska Academy, University of Gothenburg, Gothenburg, Sweden; MRC Lifecourse Epidemiology Centre, University of Southampton, Southampton, UK; NIHR Southampton Biomedical Research Centre, University of Southampton and University Hospital Southampton NHS Foundation Trust, Southampton, UK; Centre for Metabolic Bone Diseases, University of Sheffield Medical School, Sheffield, UK; Department of Medicine, School of Clinical Sciences, Monash University, Clayton, Australia; University of Manitoba, Winnipeg, Canada

**Keywords:** DXA < ANALYSIS/QUANTITATION OF BONE, osteoporosis < DISEASES AND DISORDERS OF/RELATED TO BONE, general population studies <EPIDEMIOLOGY, fracture risk assessment < PRACTICE/POLICY-RELATED ISSUES EPIDEMIOLOGY

## Abstract

Prior fracture is among the strongest predictors of future osteoporotic fracture. However, the Fracture Risk Assessment Tool (FRAX®) incorporates it as a dichotomous variable, potentially obscuring important heterogeneity related to fracture site and multiplicity. Although recent (≤2-year) fractures are recognized as conferring imminent risk, it is unclear whether non-recent high-risk fractures (nrHRFs)—specifically hip, vertebral, or multiple fractures—continue to elevate long-term risk beyond that captured by standard FRAX inputs.

In this population-based retrospective cohort study using the Manitoba Bone Mineral Density Registry linked to comprehensive provincial administrative health data, adults ≥40 years undergoing first DXA between 1996–2018 were included (N=88,653). Individuals who had a prior recent fracture (occurring less than 2 years prior to the index date) were excluded. Prior non-recent fractures (>2 years pre-index) were identified. Ten-year major osteoporotic fracture (MOF) and hip fracture probabilities were calculated using Canadian FRAX. Using Cox models adjusted for baseline FRAX probability, we evaluated residual risk associated with nrHRFs. A 2:1 train–test split was used to derive and internally validate recalibration multipliers based on observed-to-predicted fracture ratios incorporating competing risk of death.

FRAX stratified fracture risk better in those without prior nrHRF compared to those with nrHRF. In the derivation cohort, despite including prior fracture as a FRAX input, observed-to-predicted ratios indicated underestimation of MOF risk for non-recent vertebral (1.32) and multiple fractures (1.34), and underestimation of hip fracture risk for non-recent multiple fractures (1.70). Derived multipliers (×1.3 for MOF; ×1.7 for hip fracture) demonstrated good calibration in the validation cohort. Applying adjustments reclassified 13.3% and 3.5% of individuals with prior nrHRFs into high-risk MOF and hip fracture categories, respectively.

FRAX underestimates 10-year fracture risk in individuals with specific nrHRFs. Simple adjustment factors markedly improve calibration and enhance identification of patients who may benefit from fracture-preventive therapy.

## Introduction

Osteoporotic fractures represent an important global public health concern, driving substantial morbidity, mortality, and healthcare resource utilization.^1^^,^^2^ Accurate and individualized risk stratification is essential for targeted intervention strategies, particularly the appropriate use of anti-osteoporosis pharmacotherapy.^3–9^ Among the many clinical risk factors for osteoporotic fracture, prior fracture is one of the strongest predictors of future fracture.^10^ The Fracture Risk Assessment Tool (FRAX®) is the established clinical standard for estimating the 10-year absolute probability of major osteoporotic fracture (MOF) and hip fracture.^11^ While widely considered the gold standard for fracture prediction, a limitation of the current FRAX model is the incorporation of prior fracture status as a simple dichotomous variable, which does not account for the considerable heterogeneity in subsequent fracture risk imparted by the site, number, or severity of the preceding event, which can result in inaccurate fracture prediction.^10^^,^^12^^,^^13^

This limitation is particularly relevant given the recognition of “imminent fracture” risk, wherein the risk of a subsequent fracture is particularly elevated in the two years following an initial event.^14^ Risk adjustments exist for fractures that have occurred within the previous 2 years (www.fraxplus.org). We hypothesized that certain fracture types, designated non-recent high-risk fractures (nrHRF), including hip, vertebral, or multiple clinical fractures,^15–17^ occurring more than 2 years earlier confer a persistent and elevated baseline fracture susceptibility that is not fully captured by the binary “prior fracture” input in FRAX. This could lead to significant miscalibration (underestimation) of the predicted risk, resulting in the suboptimal identification of patients with heightened fracture risk who stand to benefit most from preventative care.

To address this limitation, we utilized a large, population-based cohort with comprehensive fracture and mortality data from Manitoba, Canada, to assess the calibration of FRAX in individuals with a history of nrHRF occurring more than two years prior to the index date. Following this assessment, we aimed to develop and internally validate simple, data-derived recalibration multipliers specific to different nrHRF categories (hip, vertebral and multiple fractures). The application of these adjustment factors is intended to improve the predictive accuracy of the FRAX-estimated fracture probability, thereby refining clinical decision-making for long-term fracture prevention in this high-risk patient population.

## Methods

### Study population and data sources

We conducted a retrospective cohort study of individuals residing in Manitoba, Canada. With a population of approximately 1.51 million in 2025, Manitoba is the country's fifth-most populous province.^18^ Virtually all residents access medical services through a unified public health system. All individuals residing in Manitoba are assigned a distinct personal health identification number (PHIN), which enables the linkage of their health care utilization and outcomes across various provincial administrative databases. Patient demographic information was extracted from the provincial registration database, which enumerates those eligible for health coverage in Manitoba.

Our study cohort included all individuals from the Manitoba Bone Mineral Density (BMD) Registry who underwent their initial dual-energy X-ray absorptiometry (DXA) examination between January 1996 and March 2018. This first DXA date was designated as the index date (cohort entry) for all individuals and we limited our cohort to those who were aged 40 years or older on the index date and were actively covered by the provincial health insurance program. The Manitoba BMD Program is responsible for the oversight of all clinical BMD assessments across the province, with the associated Registry functioning as the repository for all DXA results, in addition to collecting individual-level clinical characteristics. This database has been rigorously evaluated and verified for completeness with accuracy rates approaching 100%.^19^

Each subject identified in the Manitoba BMD Registry was linked to their provincial administrative health data from March 1979 to March 2018. Demographic data and dates of health insurance coverage were retrieved from the provincial registration database, which includes all individuals enrolled in Manitoba's public health insurance plan. Diagnostic codes linked to health service utilization and procedures were derived from two primary sources: physician billing claims, which utilized the International Classification of Diseases, Ninth Revision, Clinical Modification [ICD-9-CM] codes, and hospital discharge abstracts, which utilized ICD-9-CM coding before 2004, and the International Classification of Diseases, Tenth Revision, Canadian Enhancements [ICD-10-CA] after. Medication use was ascertained from the provincial pharmacy database, a repository that captures all dispensed outpatient medications.^20^ Mortality data were retrieved from the Vital Statistics registry, the official record of all births and deaths in Manitoba.^21^

The study was approved by the Health Research Ethics Board for the University of Manitoba and Manitoba’s Provincial Health Research Privacy Committee. Waiver of consent was granted by the Health Research Ethics Board for the University of Manitoba.

### Fracture assessment

We systematically identified all clinical fractures that occurred in the cohort population, excluding those of the skull, cervical region, hands, ankles and feet, using case definitions which have been previously validated against radiography.^22^^,^^23^ Fractures at the excluded sites are generally not considered to be osteoporotic fractures as they do not demonstrate expected age- or BMD-related relationships.^24–26^ Fracture events were captured between March 1979 and March 2018 using ICD-9-CM and ICD-10-CA codes recorded in both hospital discharge abstracts and physician billing claims. Importantly, our case definitions only included clinical vertebral fractures, not asymptomatic morphometric fractures, which are likely associated with different risk profiles.^27^ The incident fracture date was defined as the date of the first clinical encounter for the event, and no coding for the same fracture type was permitted in the 6-month period immediately preceding the assigned incident date. Fractures associated with high-trauma codes were excluded from the analysis.

We defined prior non-recent fracture as any clinical fracture diagnosed more than 2 years prior to the subject's index date. Prior nrHRFs included hip, vertebral and multiple fractures. Multiple prior fractures was defined as two or more clinical fractures (any fractures excluding those of skull, cervical region, hands, ankles and feet) occurring at any time more than 2 years prior to the index date. Individuals who had a prior recent fracture (occurring less than 2 years prior to the index date) were excluded, as adjustments for recent fractures already exist in FRAXplus^28^ and were the subject of a recent evaluation.^29^ Incident fractures were defined as fractures that occurred between the index date and March 2018 and were categorized as major osteoporotic fractures (MOF) if they involved the hip, vertebrae, forearm, or humerus.

### Predicted fracture risk

The 10-year absolute probability of a MOF or a hip fracture on the index date was computed for every individual using the Canadian FRAX tool (FRAX Desktop Multi-Patient Entry version 3.8), previously calibrated using nationwide Canadian hip fracture incidence and mortality rates.^30^ BMD measurements were assessed using cross-calibrated DXA devices (GE Healthcare Lunar DPX, Prodigy, iDXA; Madison, WI). A medical physicist oversaw quality control procedures and scanners demonstrated long-term stability (coefficient of variation <0.5%). Femoral neck BMD T-scores were calculated using the NHANES III white female reference values in accordance with the International Society of Clinical Densitometry guidelines.^31^ Prior fracture was ascertained as described under *Fracture assessment* above. Other clinical information required for fracture risk calculation were obtained from the Manitoba BMD Program database.^32^ Specifically, height and weight were measured at the time of DXA. Parental hip fracture, smoking status, high alcohol intake, rheumatoid arthritis and secondary causes of osteoporosis were ascertained from the intake questionnaire completed at the time of DXA. To ensure comprehensive data for all individuals, the questionnaire responses were supplemented and corroborated using data from the previously described linked provincial administrative health databases as previously described.^33^ Oral glucocorticoid exposure greater than 90 days in the year prior to the index date were ascertained using the provincial pharmacy database.

### Statistical analyses

Summary statistics were used to describe the baseline characteristics of the full cohort and stratified by prior nrHRF status. Using Cox proportional hazards models, we estimated the hazard ratios (HRs) and 95% confidence intervals (CIs) for incident MOF and incident hip fracture for each standard deviation (SD) increase in MOF and hip fracture probability, respectively, for each type of prior nrHRF. Baseline fracture probabilities were log-transformed to account for a skewed distribution. The proportional hazards assumption was confirmed graphically from the log-log survival plots. To examine for effects of prior nrHRF beyond the inclusion of prior fracture in FRAX for predicting incident MOF and hip fracture, we adjusted for baseline FRAX probability (which included BMD and prior fracture as an input). As a secondary analysis, we also estimated HRs for incident MOF and incident hip fracture per SD decrease in BMD. We also estimated HRs for incident MOF and incident hip fracture per SD increase in FRAX not including BMD for each type of prior nrHRF.

To develop recalibration multipliers, we used a train-test approach by randomly splitting our cohort into a derivation subset (⅔) and an internal validation subset (⅓). In the derivation subset, we estimated the cumulative incidence function for MOF and hip fracture, stratified by prior nrHRF and taking into consideration the competing risk of death.^34^ The 10-year observed fracture probability divided by FRAX-predicted 10-year fracture probability was used to estimate recalibration multipliers for MOF and hip fracture prediction in those with prior nrHRF. These recalibration multipliers were then applied to the FRAX estimates for the internal validation subset and calibration was re-assessed by comparing the observed and adjusted FRAX-predicted fracture probabilities. We also assessed the number of individuals in the internal validation subset who were reclassified into the high-risk category (10-year MOF risk ≥20% and hip fracture risk ≥3%. We tested for effect modification by sex (female vs male) and age category (<65 vs >65 years) using stratified analysis. Statistical analyses were performed with SPSS for Windows (Version 30.0) and p-values <0.05 were considered statistically significant.

## Results

There was a total of 88,653 individuals in the cohort, with a mean age of 64.6 years of which 90.3% were female; 17,881 (20.2%) had a prior non-recent fracture of which 6016 (6.8%) fell within one of the nrHRF categories (Table 1). Of those with prior nrHRF, there were 1,455 (1.6%) with hip fracture, 3,293 (3.7%) with vertebral fracture and 1,622 (1.8%) with multiple fractures. Over an average observation period of 9.0 years, 9060 (10.2%) individuals suffered an incident MOF and 3067 (3.5%) suffered an incident hip fracture.

**Table 1:** Cohort characteristics and fracture outcomes, by prior non-recent high risk fracture status.

	Full Cohort N = 88,653	Prior non-recent high risk fracture			
		None N = 82,637	Hip N = 1455	Vertebral N = 3293	Multiple N = 1622
**Baseline characteristics**					
**Age (years)**	64.6 ± 11.0	64.3 ± 10.8	73.5 ± 10.9	67.0 ± 11.9	68.4 ± 11.4
**Sex (female)**	80,018 (90.3)	75,125 (90.9)	1183 (81.3)	2565 (77.9)	1436 (88.5)
**Femoral neck T-score**	−1.4 ± 1.0	−1.3 ± 1.0	−2.5 ± 0.8	−1.7 ± 1.0	−2.0 ± 0.9
**MOF probability %**	10.1 ± 7.1	9.5 ± 6.5	23.1 ± 10.8	16.0 ± 9.4	18.7 ± 10.1
**Hip fracture probability %**	2.4 ± 3.9	2.1 ± 3.5	8.8 ± 8.1	4.5 ± 5.7	5.8 ± 6.8
**Incident fractures**					
**Observation (years)**	9.0 ± 5.5	9.1 ± 5.5	7.0 ± 5.1	8.1 ± 5.4	7.4 ± 5.1
**MOF**	9060 (10.2)	7867 (9.5)	279 (19.2)	677 (20.6)	340 (21.0)
**Hip fracture**	3067 (3.5)	2620 (3.2)	142 (9.8)	206 (6.3)	146 (9.0)

Values are expressed as number ± SD or number (%). MOF, major osteoporotic fracture.

HRs for incident MOF and incident hip fracture per SD increase in fracture probability were highest in those with no prior nrHRF, and still significant but slightly weaker after prior nrHRF (Table 2). Overall, the HRs were larger for each SD increase in FRAX than for each SD decrease in BMD. Prior non-recent vertebral fracture had an adjusted HR (aHR) of 1.41 (95% CI 1.28-1.56) and prior non-recent multiple fractures had an aHR of 1.41 (95% CI 1.23-1.61) for incident MOF. Prior non-recent multiple fractures also showed a significantly elevated aHR for incident hip fracture [aHR 1.41 (95% CI 1.15-1.72). These significant aHR demonstrated residual risk unaccounted for by FRAX-derived fracture probability. After adjusting for baseline fracture risk, the aHR for incident MOF and incident hip fracture were non-significant for those with prior non-recent hip fracture compared with no prior nrHRF (Table 3). Multiple testing did not show significant effect modification from the different nrHRF subgroups when stratified by sex (female vs male) or age (<65 vs >65 years) (with all Bonferroni-adjusted p-values >0.1). HRs for incident MOF and incident hip fracture per SD decrease in BMD were highest in those with no prior nrHRF, but BMD was remained a significant predictor of MOF (HR range 1.34 to 1.39) and hip fracture (HR range 1.48 to 1.81) in all categories of nrHRF (Supplementary eTable S1). HRs for incident MOF and incident hip fracture per SD increase in FRAX not including BMD were overall lower than when BMD was included in FRAX (Supplementary eTable S2); and once adjusted for baseline fracture probability, the aHR were larger than when BMD was included in FRAX (Supplementary eTable S3).

**Table 2:** Hazard ratios (HR) and 95% confidence intervals (CI) for incident fracture per standard deviation (SD) increase in fracture probability in the derivation subset, N = 58,904.

	Incident MOF: HR per SD MOF probability	Incident hip fracture: HR per SD hip fracture probability
**No prior nrHRF**	1.78 (1.73-1.83)	3.38 (3.18-3.59)
**Prior hip fracture**	1.42 (1.16-1.74)	2.18 (1.49-3.19)
**Prior vertebral fracture**	1.50 (1.35-1.66)	2.25 (1.79-2.82)
**Prior multiple fractures**	1.42 (1.21-1.67)	2.22 (1.66-2.99)

MOF, major osteoporotic fracture.

**Table 3:** Adjusted hazard ratios (aHR) and 95% confidence intervals (CI) for incident fracture, adjusted for baseline fracture probability in the derivation subset, N=58,904.

	Incident MOF: aHR (95% CI)^*^	Incident hip fracture: aHR (95% CI)^**^
**No prior nrHRF**	Reference	Reference
**Prior hip fracture**	0.97 (0.84-1.13)	0.98 (0.79-1.21)
**Prior vertebral fracture**	1.41 (1.28-1.56)	1.03 (0.87-1.22)
**Prior multiple fractures**	1.41 (1.23-1.61)	1.41 (1.15-1.72)

^*^Adjusted for MOF probability

^**^Adjusted for hip fracture probability.

The cohort was then split into a ⅔ derivation subset (N = 58,904) and ⅓ validation subset (N = 29,964). Cohort characteristics and fracture outcomes of derivation and validation subsets were similar (Supplementary eTable S4). We compared the 10-year observed-to-predicted FRAX-predicted MOF and hip fracture probabilities in both the derivation and internal validation subsets (Table 4, Figure 1). In the derivation subset, significantly increased ratios for MOF were seen after prior non-recent vertebral fracture (ratio 1.32, 95% CI 1.19-1.44) and after prior non-recent multiple fractures (ratio 1.34, 95% CI 1.17-1.51). From these, we derived the adjustment factor of x1.3 for predicted MOF risk in the setting of either non-recent prior vertebral fracture or multiple fractures. When this adjustment factor was applied to the internal validation subset, the observed-to-predicted ratio for prior non-recent vertebral fracture was 1.09 (95% CI 0.96-1.23) and for multiple fractures was 0.93 (95% CI 0.75-1.10), demonstrating good calibration for MOF prediction.

**Table 4:** Ratios of observed and predicted 10-year fracture probability (with and without adjustment^*^), with 95% confidence interval (95% CI), by prior non-recent high risk fracture (nrHFR), in the derivation subset (N=58,904) and internal validation subset (N = 29,964).

Prior nrHRF	MOF probability: Observed/predicted ratio (95% CI)				Hip fracture probability: Observed/predicted ratio (95% CI)			
	Derivation subset		Validation subset		Derivation subset		Validation subset	
	Derivation	Adjusted^*^	Unadjusted	Adjusted^*^	Derivation	Adjusted^*^	Unadjusted	Adjusted^*^
**Hip**	0.90 (0.77-1.03)	–	0.92 (0.74-1.11)	–	1.17 (0.91-1.43)	–	1.14 (0.79-1.50)	–
**Vertebral**	1.32 (1.19-1.44)	1.09 (0.96-1.23)	1.42 (1.24-1.60)	1.09 (0.96-1.23)	1.16 (0.92-1.41)	–	1.24 (0.88-1.61)	–
**Multiple**	1.34 (1.17-1.51)	0.93 (0.75-1.10)	1.21 (0.98-1.44)	0.93 (0.75-1.10)	1.70 (1.33-2.07)	0.99 (0.66-1.31)	1.68 (1.12-2.23)	0.99 (0.66-1.31)

^*^Adjustment: x1.3 for MOF; x1.7 for hip fracture. MOF, major osteoporotic fracture. – denotes absence of adjustment as there was no significant difference between observed and predicted fracture risk seen in the derivation cohort.

**Figure 1:**
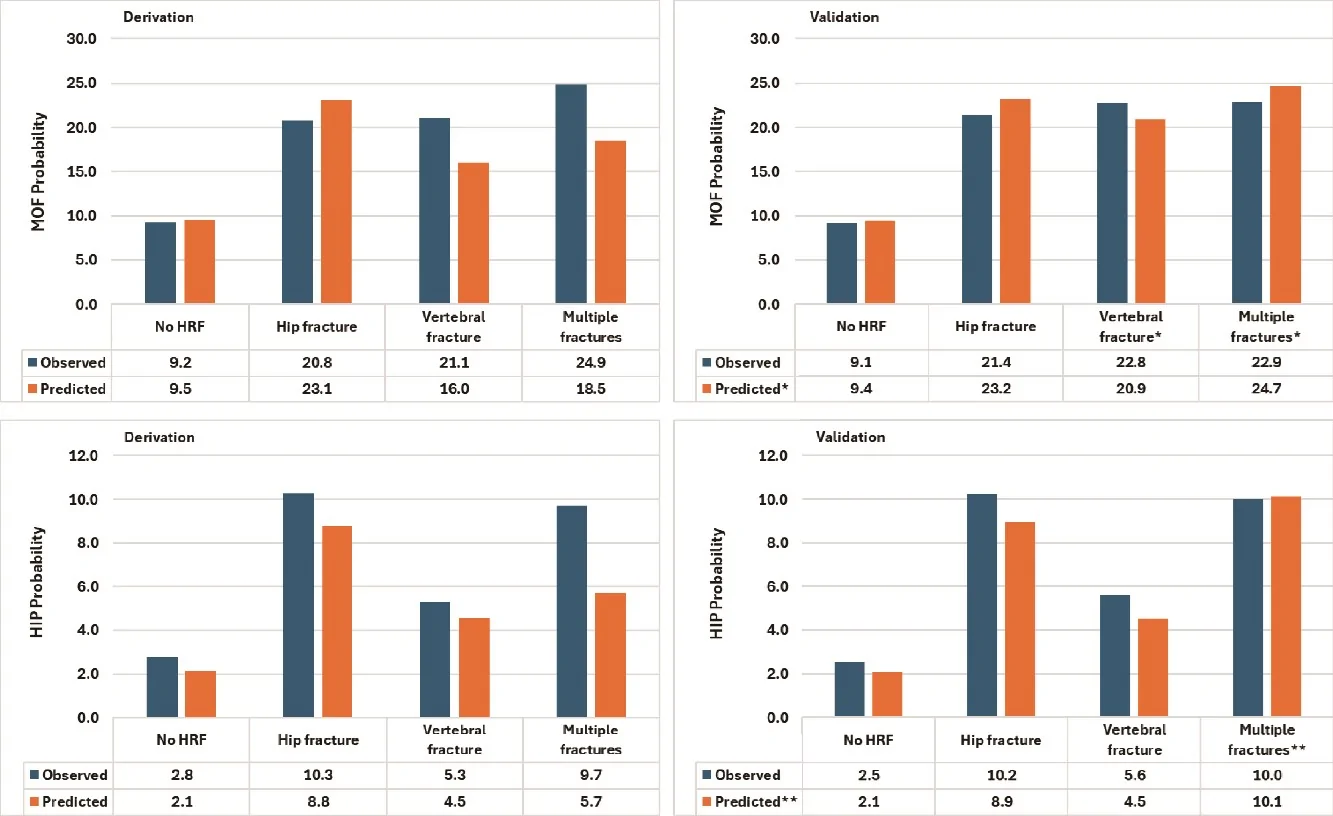
Observed versus unadjusted FRAX-predicted 10-year major osteoporotic fracture (MOF) and hip fracture percent probability for the derivation subset, with observed versus adjusted FRAX-predicted probability for the internal validation subset (^*^ x1.3 for MOF; ^**^ x1.7 for hip fracture).

In the derivation subset, the 10-year observed-to-predicted ratio for hip fracture was significantly elevated in those with prior non-recent multiple fractures (ratio 1.70, 95% CI 1.33-2.07, Table 4). From this, we derived the adjustment factor of x1.7 for predicted hip fracture risk in the setting of non-recent prior multiple fractures. When this adjustment factor was applied to the internal validation subset, the observed-to-predicted ratio for prior non-recent multiple fractures was 0.99 (95% 0.66-1.31), demonstrating good calibration for hip fracture prediction.

Of the 2032 individuals in the internal validation subset who had a prior nrHRF, 270 (13.3%) were reclassified from <20% to > 20% risk of MOF and 72 (3.5%) were reclassified from <3% to >3% risk for hip fracture. Among those with predicted MOF risk <20% who experienced an incident MOF during follow up, 65 of 216 (30.0%) had their MOF risk reclassified to >20%. For those with predicted hip fracture risk <3% who experienced an incident hip during follow up, 5 of 19 (26.3%) had their hip fracture risk reclassified to >3%.

## Discussion

In this retrospective cohort study, we found that while FRAX stratified risk in all of the nrHRF subgroups, the prior fracture variable in FRAX does not fully account for the risk of nrHRFs, with significant underestimation of MOF risk in those with non-recent clinical vertebral fracture and multiple fractures, and underestimation of hip fracture risk in those with non-recent multiple fractures. With the application of simple adjustment factors (Box 1), calibration was restored and resulted in a clinically meaningful proportion of those with nrHRFs being reclassified into a high risk fracture category.

Based on data from the Reykjavik Study fracture register which showed underestimation of fracture risk for those with fractures occurring within the previous 2 years, adjustment ratios were developed for recent fractures.^35^ Recognition that the risk of a recurrent fragility fracture is particularly high immediately following the fracture, FRAXplus® provides adjustments to FRAX®-based fracture probabilities accounting for the site (vertebral, hip, humerus, forearm) of a recent fracture.^28^ Similarly, it is widely accepted that multiple prior fractures increase the risk of future fractures^36^^,^^37^ and accordingly, FRAXplus provides an option to adjust for history of multiple fractures.^28^

Previous studies have shown that in addition to the number and recency of past fractures, the site of past fractures modifies future fracture risk,^36^ with prior hip, and vertebral fractures conferring the highest risk of future fractures. In fact, guidelines frequently assign individuals automatically to the high risk fracture category, meeting treatment threshold, in the presence of prior hip or vertebral fractures, irrespective of other clinical risk factors, including BMD.^38^ A previous study showed that FRAX significantly underestimated fracture risk in those with a vertebral fracture diagnosed on a vertebral fracture assessment (VFA) by 50 to 75%.^12^ Interestingly, in our study, FRAX prediction in the setting of non-recent prior hip fracture performed well. This could be due to this subgroup being the oldest, with the lowest BMD and the highest baseline FRAX risk-factors which likely saturate risk stratification. Further, evaluating non-recent fractures results in survivorship bias of those who survived more than 2 years after hip fracture, likely excluding the frailest hip fracture sufferers.

Box 1. Adjustment factors for non-recent high risk fracture

**Table:** 

**MOF probability**	
**• Prior vertebral fracture** ** • Prior multiple fracture**	• Multiply FRAX MOF probability by 1.3
**Hip fracture probability**	
** • Prior multiple fracture**	• Multiply FRAX hip fracture probability by 1.7

A primary strength of this study lies in the utilization of a large, population-based cohort from the Manitoba BMD Registry, which captures nearly all DXA scans performed in the province, along with thorough data on clinical risk factors. Given that virtually all Manitoba residents access medical services through a unified public health system, we had comprehensive linkage for fracture ascertainment. Further, we used fracture case definitions which have previously been validated against radiographically confirmed fractures in the province.^39^ The robust methodology, including the train-test approach with derivation and validation subsets to develop and internally validate the recalibration multipliers, strengthens confidence in the derived adjustment factors.

The study's limitations include its retrospective design, which limits our detection of fractures to those that were clinically diagnosed or assessed by a physician. Although the case definitions for fracture were systematically applied and previously validated, vertebral fractures that did not result in a clinical diagnosis would have been missed. Furthermore, while the cohort is derived from a population-based BMD registry, it is restricted to individuals who underwent a DXA scan, leading to an inherent selection bias towards a higher-risk population compared to the general public. However, as this study specifically examines those with prior HRFs, the selection bias likely aligns with the population of interest. Importantly, the study cohort was also composed mostly of females (approximately 90%), which limits the generalizability of the findings and derived adjustment factors, particularly for estimating fracture risk in the male population. Finally, while the study effectively addressed nrHRFs, the adjustment factors are specific to the Canadian FRAX tool and were derived from a single population, meaning they require external validation before being applied broadly to other cohorts or FRAX models.

In conclusion, this study identified significant underestimation of future fracture risk in individuals with some types of nrHRFs and successfully derived and internally validated recalibration multipliers to account for the persistent, elevated fracture risk from nrHRF, a limitation of the current FRAX and FRAXplus tools. These adjustment factors were shown to significantly improve model calibration, and resulted in a substantial reclassification of individuals into a high-risk category, which has significant and immediate implications for initiating anti-osteoporosis treatment and potentially improving patient outcomes. The results of this study may also help to inform future fracture prediction tools.

## Supplementary Material

nrHRF_supp_zjag049

## Data Availability

Data sharing is not permitted under the Researcher Agreement with Manitoba Health and Seniors Care (MHASC). However, researchers may apply for data access through the Health Research Ethics Board for the University of Manitoba and the Health Information and Privacy Committee of MHASC.
