# Supplementary material for: Predicting fracture risk after non-recent high risk fracture: improving accuracy with simple modifiers to FRAX
Source: J Bone Miner Res. 2026 Mar 11;41(11):1241–8. doi: 10.1093/jbmr/zjag049 (PMC13716914; doi:10.1093/jbmr/zjag049)
Supplement: Supplementary file 1 [file nrhrf_supp_zjag049.docx]

**SUPPLEMENTARY MATERIALS**

**eTable 1. Hazard ratios (HR) and 95% confidence intervals (CI) for incident fracture per standard deviation (SD) decrease in bone mineral density (BMD) in the derivation subset, N=58,904**

|  | Incident MOF:  HR per SD BMD decrease | Incident hip fracture:  HR per SD BMD decrease |
| --- | --- | --- |
| No prior nrHRF | 1.76 (1.71-1.81) | 2.54 (2.41-2.68) |
| Prior hip fracture | 1.34 (1.11-1.61) | 1.48 (1.14-1.93) |
| Prior vertebral fracture | 1.39 (1.26-1.52) | 1.80 (1.52-2.15) |
| Prior multiple fractures | 1.39 (1.20-1.62) | 1.81 (1.43-2.29) |

*MOF, major osteoporotic fracture.*

**eTable 2. Hazard ratios (HR) and 95% confidence intervals (CI) for incident fracture per standard deviation (SD) increase in fracture probability, not considering bone mineral density, in the derivation subset, N=58,904**

|  | Incident MOF:  HR per SD MOF probability | Incident hip fracture:  HR per SD hip fracture probability |
| --- | --- | --- |
| No prior nrHRF | 1.64 (1.60-1.69) | 2.75 (2.60-2.90) |
| Prior hip fracture | 1.41 (1.15-1.73) | 2.17 (1.54-3.04) |
| Prior vertebral fracture | 1.46 (1.31-1.64) | 2.11 (1.69-2.64) |
| Prior multiple fractures | 1.38 (1.17-1.63) | 2.07 (1.57-2.73) |

*MOF, major osteoporotic fracture.*
**eTable 3. Adjusted hazard ratios (aHR) and 95% confidence intervals (CI) for incident fracture, adjusted for baseline fracture probability, not considering BMD, in the derivation subset, N=58,904**

|  | Incident MOF:  aHR (95%CI)* | Incident hip fracture:  aHR (95%CI)** |
| --- | --- | --- |
| No prior nrHRF | Reference | Reference |
| Prior hip fracture | 1.16 (1.00-1.35) | 1.20 (0.97-1.48) |
| Prior vertebral fracture | 1.50 (1.36-1.66) | 1.06 (0.90-1.26) |
| Prior multiple fractures | 1.58 (1.38-1.80) | 1.56 (1.28-1.91) |

** Adjusted for MOF probability; ** Adjusted for hip fracture probability.*

**eTable 4. Cohort characteristics and fracture outcomes of derivation and validation subsets**

|  | **Derivation Subset**  **N = 58,904** | **Validation Subset**  **N = 29,964** |
| --- | --- | --- |
| **Baseline characteristics** | | |
| Age (years) | 64.6 ± 11.0 | 64.5 ± 10.9 |
| Sex (female) | 53,232 (90.4) | 26,977 (90.0) |
| Prior fracture | 11,863 (20.1) | 6026 (20.1) |
| Prior high risk fracture | 3984 (6.8) | 2032 (6.8) |
| Femoral neck T-score | -1.4 ± 1.0 | -1.4 ± 1.0 |
| MOF probability % | 10.1 ± 7.2 | 10.0 ± 7.1 |
| Hip fracture probability % | 2.4 ± 3.9 | 2.3 ± 3.8 |
| **Incident fractures** | | |
| Observation (years) | 9.0 ± 5.5 | 9.0 ± 5.5 |
| MOF | 6061 (10.3) | 2999 (10.0) |
| Hip fracture | 2079 (3.5) | 992 (3.3) |

*Values are expressed as number ± SD or number (%). MOF, major osteoporotic fracture.*
